# Braithwaite’s Retrospect of Practical Medicine and Surgery—for the Half-Year Ending January, 1864

**Published:** 1864-03

**Authors:** 


Braithwaite's Retrospect of Practical Medicine and Surgery—For the Half-Year Ending January, 1864. Uniform American Edition. Published by W. A. Townsend, 39 Walker street, New York.

The great convenience and value of this semi-annual summary of medical literature is too well known to the profession to require any comments from us.

Price $2 per annum in advance.

